# Desiccation stress and tolerance in green algae: consequences for ultrastructure, physiological and molecular mechanisms

**DOI:** 10.3389/fpls.2013.00327

**Published:** 2013-08-22

**Authors:** Andreas Holzinger, Ulf Karsten

**Affiliations:** ^1^Functional Plant Biology, Institute of Botany, University of InnsbruckInnsbruck, Austria; ^2^Applied Ecology and Phycology, Institute of Biological Sciences, University of RostockRostock, Germany

**Keywords:** aeroterrestrial algae, cell wall, dehydration, desiccation tolerance, phylogeny of green algae, osmolyte, soluble carbohydrates, turgor pressure

## Abstract

Although most green algae typically occur in aquatic ecosystems, many species also live partly or permanently under aeroterrestrial conditions, where the cells are exposed to the atmosphere and hence regularly experience dehydration. The ability of algal cells to survive in an air-dried state is termed desiccation tolerance. The mechanisms involved in desiccation tolerance of green algae are still poorly understood, and hence the aim of this review is to summarize recent findings on the effects of desiccation and osmotic water loss. Starting from structural changes, physiological, and biochemical consequences of desiccation will be addressed in different green-algal lineages. The available data clearly indicate a range of strategies, which are rather different in streptophycean and non-streptophycean green algae. While members of the Trebouxiophyceae exhibit effective water loss-prevention mechanisms based on the biosynthesis and accumulation of particular organic osmolytes such as polyols, these compounds are so far not reported in representatives of the Streptophyta. In members of the Streptophyta such as *Klebsormidium*, the most striking observation is the appearance of cross-walls in desiccated samples, which are strongly undulating, suggesting a high degree of mechanical flexibility. This aids in maintaining structural integrity in the dried state and allows the cell to maintain turgor pressure for a prolonged period of time during the dehydration process. Physiological strategies in aeroterrestrial green algae generally include a rapid reduction of photosynthesis during desiccation, but also a rather quick recovery after rewetting, whereas aquatic species are sensitive to drying. The underlying mechanisms such as the affected molecular components of the photosynthetic machinery are poorly understood in green algae. Therefore, modern approaches based on transcriptomics, proteomics, and/or metabolomics are urgently needed to better understand the molecular mechanisms involved in desiccation-stress physiology of these organisms. The very limited existing information is described in the present review.

## INTRODUCTION

Water is essential for all organisms on Earth, and thus the removal of water from an algal cell represents a severe, often lethal stress. The structure of intracellular biomolecules and membranes is maintained by water molecules, and thus dehydration leads to an often irreversible aggregation of macromolecules and disintegration of organelles. Although dehydration tolerance and desiccation tolerance are often cited as synonymous mechanisms, they must be clearly distinguished. Desiccation, the equilibration of an organism to the relative humidity of the surrounding atmosphere, is an intense stress factor that in most phototrophic organisms produces high mortality. Nevertheless, some plant species are highly tolerant to desiccation and possess mechanisms to maintain and protect cellular integrity and to repair any concomitant damage. Desiccation tolerance can be defined as the ability to survive drying to about 10% remaining water content, which is roughly equivalent to 50% relative air humidity (RH) at 20°C (=water potential of - 100 MPa; Alpert, 2006; Oliver et al., 2010). Poikilohydric plants such as lichens and algae cannot actively regulate their water content (Kranner et al., 2008), which can easily lead to desiccation under water-limited conditions. In contrast, homoiohydric plants possess mechanisms to regulate their water status, which may enable them to tolerate drying. The two types of tolerance differ fundamentally. Dehydration-tolerance mechanisms always involve maintaining homeostasis during dehydration as long as possible by actively regulating the water status, and hence minimizing or repairing any damage as fast as possible. In contrast, desiccation tolerance consists of strategies to survive the complete loss of water.

Desiccation tolerance is well documented in the literature for cyanobacteria and lichens (e.g., Lange et al., 1989; Potts, 1994, 2001; Kranner et al., 2003, 2008; Potts et al., 2005). Other prominent examples of eukaryotes are mosses, which can survive full desiccation for long periods of time (e.g., Oliver and Bewley, 1997; Proctor, 2000; Proctor and Smirnoff, 2000; Oliver et al., 2004; Proctor et al., 2007; Pressel et al., 2009; Pressel and Duckett, 2010). However, the actual number of moss species that can tolerate desiccation is still under discussion. While Alpert (2006) stated that most moss species tested are desiccation-tolerant, Wood (2007) reported that only about 1% of all known bryophytes have been experimentally proven to possess vegetative desiccation tolerance. Angiosperm plant species with the capacity to allow desiccation in their vegetative organs (such as leaves) are rare (e.g., Moore et al., 2009; Gaff and Oliver, 2013). These specialist plants have been termed “resurrection plants” and rely on the induction of mechanisms to protect cellular integrity during water loss, although a certain time interval is required to induce this tolerance (Oliver and Bewley, 1997; Cooper and Farrant, 2002; Oliver et al., 2010). Lichens, mosses, and angiosperm resurrection plants are well studied concerning desiccation tolerance (chapters in this Research Topic on desiccation tolerance in Frontiers in Plant Science), and even transcriptome analyses of desiccated or rehydrated resurrection plants and mosses exist (e.g., Oliver et al., 2004; Rodriguez et al., 2010; Gechev et al., 2013). In contrast, for green algae only a few publications on the physiological effects of desiccation are available. Some green algae implement structural, physiological, biochemical or molecular mechanisms to survive severe water deficit. Desiccation tolerance of some green algae has been described nearly 100 years ago (e.g. Piercy, 1917). “The topic of desiccation tolerance in algae is a fertile field for research” stated Bewley, nearly 35 years ago (Bewley, 1979), yet, to date only a few studies have dealt with this topic. Davis (1972) reported that especially in green algae, desiccation tolerance is well represented: among 72 genera (119 species) of green algae, 17 genera (25 species) have desiccation-tolerant vegetative cells, 11 genera (19 species) have desiccation-tolerant reproductive cells, and 26 genera (29 species) have desiccation-tolerant cysts and resting cells. However, the underlying mechanisms of desiccation tolerance in green algae, and the components and pathways that facilitate them are only partly understood, and hence in this review we will describe and discuss the current knowledge of desiccation stress in green algae.

## DESICCATION IN AQUATIC GREEN ALGAE

**FIGURE 1:**
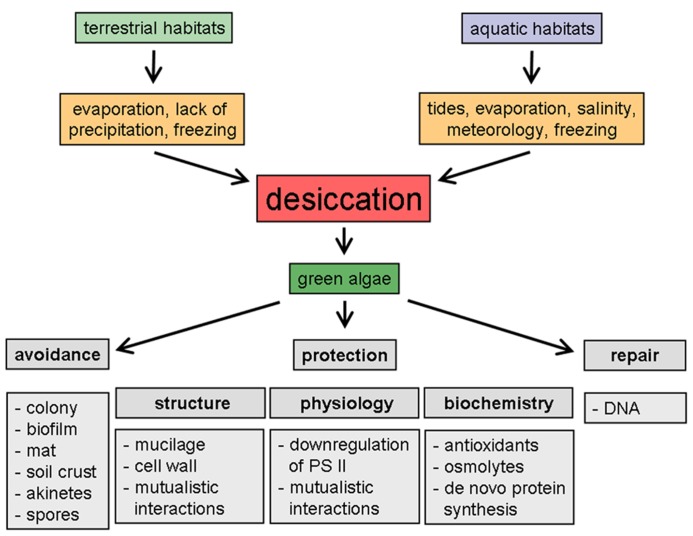
**Schematic representation of desiccation-induced phenomena in green algae from different habitats**.

When considering dehydration in green algae, it is essential to first distinguish between aquatic (marine and freshwater) and terrestrial habitats. The effects of dehydration on green algae from different habitats are presented schematically in **Figure 1**. The life form may strongly affect desiccation tolerance in these organisms, because they can be distinguished based on their size into micro- and macro-algae, with the largest specimens found in the ocean. While many green microalgae live in ephemeral freshwater ponds, marine green macroalgae such as seaweeds occur in the intertidal or supralittoral zone along rocky coasts, where they preferentially grow as sessile organisms attached to hard substrata such as rocks, gravel, or coral reefs, or as epiphytes on salt-marsh plants, mangroves, and mussel colonies. Here they are mainly confronted with dehydration when exposed at low tides. In contrast, ephemeral ponds may experience desiccation at regular and much longer intervals and for much longer periods (e.g., Evans, 1958). Consequently, dehydration during exposure at low tide in the marine environment is typically a local and “short-term” rather than a global and “long-term” factor, although it can be highly variable in coastal regions due to neap tides and meteorological conditions. In addition, dehydration in marine green algae can also be mediated by hypersaline conditions, due to tidal flows, hydrology, wind, and evaporation, or by freezing. Salinity stress and desiccation are different types of water deprivation. Whereas under hypersaline conditions, seaweed cells are still in full contact with liquid water of decreased water potential, desiccation leads to more intense cellular dehydration. Therefore, salinity stress is often defined as “physiological drought” (Kirst, 1990). Because hypersalinity and desiccation affect the internal osmotic potential, which is essential for maintaining turgor pressure as the driving force for growth, the acclimation responses of marine green algae are comparable (for a review of osmotic acclimation, see Karsten, 2012). Although both types of stresses result in a reduction of the cellular water potential, there is one fundamental difference. During desiccation, cellular ionic concentrations increase, but the ion ratios remain constant. In contrast, during salinity stress, algal cells may not only increase their ionic concentrations, but also undergo changes in ion ratios owing to selective uptake. This must be taken into account when comparing the results obtained with green algal species under salt or desiccation stress. However, and as mentioned before, in the marine environment the intensity of dehydration is closely connected to the tidal rhythm, as the next high tide will allow the algae to recover.

## DESICCATION IN AEROTERRESTRIAL GREEN ALGAE

**FIGURE 2:**
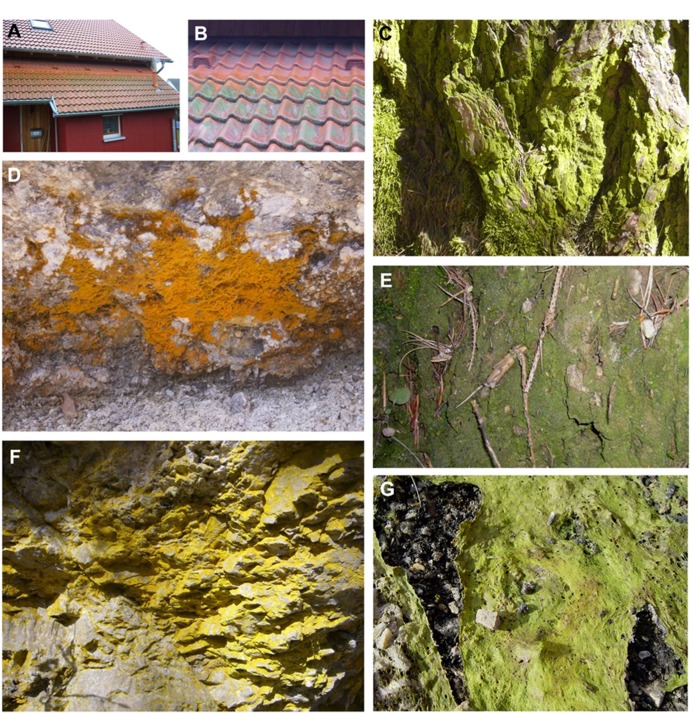
**Different habitats of desiccation-tolerant green algae, on anthropogenic (A,B) or natural (C–G) surfaces.** **(A)** Aeroterrestrial biofilm dominated by *Apatococcus* sp., growing on roof tiles in Rostock, Germany, **(B)** detail from the same roof, note the biofilm only on the rain-exposed tiles, **(C)** *Apatococcus* sp. on bark of tree, Innsbruck, Tyrol, **(D)** *Trentepohlia* sp. on rock surface, Innsbruck, Tyrol, **(E)** Biological soil crust dominated by *Klebsormidium* sp. and *Stichococcus* sp., pine forest near Innsbruck, **(F)** Epilithic green algae on shaded rock surface, Tulfes, Tyrol, **(G)** *Zygnema* sp. mat near Ny Alesund, Svalbard (**G** reprinted from Holzinger et al., 2009 with permission from Elsevier).

Although most green algae typically inhabit aquatic environments, many taxa occur on land, where they participate in symbiotic associations with fungi, forming lichens (e.g., Ettl and Gärtner, 1995; Friedl, 1997; Friedl and Rybalka, 2012), or grow on man-made surfaces such as roof tiles (**Figures 2A,B**) or on natural surfaces (**Figures 2C–G**) such as tree bark (Rindi and Guiry, 2004; Karsten et al., 2007). In addition, many green algal taxa can grow under rocks (hypolithic) or on the surface (epidaphic, **Figures 2D,F**) or just below the surface (endedaphic) of the soil (Singh, 1941; Friedmann et al., 1967; Bell, 1993). Green algae are also typical components of so-called biological soil crusts (**Figure 2E**), which are found on all continents on Earth, in arid and semi-arid regions (e.g., Flechtner, 2007) as well as from tropical to polar regions, in the high alpine zone, i.e., in habitats where soil moisture is limiting and vascular plant cover is sparse (e.g., Fritsch and Haines, 1923; Belnap and Lange, 2001). Aeroterrestrial algae form an intimate association with soil particles, which exists within, or immediately on top of, the uppermost millimeters of soil (Nienow, 1996; Belnap and Lange, 2001; Büdel, 2005; Cardon et al., 2008; and references therein). There, the biological crusts form water-stable aggregates that have important ecological roles in primary production, nutrient cycling, water retention, and stabilization of soils (Evans and Johansen, 1999; Lewis, 2007).

Lüttge and Büdel (2010) reported on red to purple-brown layers of *Trentepohlia umbrina* (Ulvophyceae) on trees with rough bark, whereas the smooth bark of other trees was covered by green biofilms composed of *Desmococcus *sp., *Apatococcus *sp., *Trebouxia *sp. and *Coccomyxa *sp.. Other terrestrial habitats for green algae include the atmosphere (airborne algae; Sharma et al., 2007), caves (Mulec and Kosi, 2009; and references therein) and spider webs (Azúa-Bustos et al., 2010). Five taxa of green soil algae showed viable and growing cells even after 35 years preservation of the soil sample (Trainor and Gladych, 1995), and *Protosiphon botryoides* survived for 43 years (Lewis and Trainor, 2012).

Compared to aquatic environments, aeroterrestrial green algae are exposed to harsher environmental conditions, such as great differences in water potential between the terrestrial habitat (e.g., soil) and the atmosphere, resulting in regular desiccation leading to dehydration of the cells. In alpine regions of Europe, for example, water availability frequently fluctuates, from fluid droplets after rain or snow, to extended periods of dryness or freezing. Water availability, which includes precipitation, condensation, and water vapor, is therefore the key ecological prerequisite for long-term survival of aeroterrestrial algae in such habitats, because only fully hydrated and ultrastructurally intact cells will be able to function physiologically (Gray et al., 2007; Karsten et al., 2007, 2010; Holzinger, 2009; Karsten and Holzinger, 2012). In terrestrial ecology, drought is defined as an extended period of months or years when a region undergoes a deficiency in its water supply, such as lack of precipitation. Although drought is a normal, recurring feature of the climate in many parts of the world, global-change scenarios predict intensified desertification (Brito et al., 2013), which of course will have a substantial impact on the terrestrial ecosystems and organisms of the affected region.

## PHYLOGENETIC RELATIONSHIPS IN GREEN ALGAE

In the previous sections, we distinguished between green algae from aquatic and aeroterrestrial habitats, which are ecologically interesting in terms of the intensity and duration of dehydration in the different habitats. Büdel (2011) provided a recent overview of desiccation-tolerant eukaryotic algae, which included many green-algal taxa. However, he did not relate this tolerance to their phylogenetic positions and relationships, which could be important to better understand the strategies developed in the different lineages.

Green algae are a morphologically and ecologically diverse monophyletic lineage of the Archaeplastida, and belong to different clades that are considered as “subphyla,” namely the Chlorophyta and the Streptophyta (Lewis and McCourt, 2004; Becker and Marin, 2009; Timme and Delwiche, 2010; Wodniok et al., 2011; Friedl and Rybalka, 2012; Leliaert et al., 2012; Timme et al., 2012; Becker, 2013). It is far beyond the scope of this review to give a detailed description of the phylogenetic and molecular evolutionary relationships among green algae, and hence the reader is referred to recent excellent overviews for details (e.g., Friedl and Rybalka, 2012; Leliaert et al., 2012). Here, we highlight the most important lineages that have an aeroterrestrial lifestyle.

The split between Chlorophyta and Streptophyta, the main groups of the green-algal lineage, took place more than 900 million years ago (Mya). For example, the time tree of life () gives the time of divergence between *Ulva* (Chlorophyta) and *Klebsormidium* (Streptophyta) as a mean of 976.3 Mya. Becker (2013) drew an interesting correlation between the upper limit of the divergence time of the Streptophyta/Chlorophyta, which coincides with the Cryogenian geologic period when the Earth was covered with snow and ice (“hard snowball stage” of the earth) and a dramatic decrease of atmospheric carbon. This resulted in different physiological properties of the two lineages (e.g., photorespiration pathways: Stabenau and Winkler, 2005; occurrence of special photoprotection mechanisms: Gerotto and Morosinotto, 2013; see below), which might also have influenced their ability to tolerate desiccation.

Among the Chlorophyta, one can distinguish between “Prasinophytes” and “core Chlorophytes,” the latter containing the classes Trebouxiophyceae, Chlorophyceae, and Ulvophyceae (Leliaert et al., 2012). Whereas within the Prasinophytes virtually no aeroterrestrial forms occur, in the Trebouxiophyceae (“lichen algae group”) the terrestrial lifestyle is common (e.g., Lewis and Lewis, 2005; Darienko et al., 2010; Friedl and Rybalka, 2012). Most of the presently known members of this class are coccoid unicells or colonial coccoids, and many of them form lichens (e.g., *Trebouxia *sp., *Asterochloris* sp.). Terrestrial forms such as *Fritschiella *sp. are also known in the Chlorophyceae (order Chaetophorales).

Among the Ulvophyceae, members of the Genotypus *Ulva* are widely distributed, abundant marine macroalgae, living in the intertidal zone and hence tolerating periodic desiccation by special physiological adaptation (Zou et al., 2007; Gao et al., 2012). The Trentepohliales is an entirely terrestrial order within the Ulvophyceae, and its members form filaments of uninucleate cells, which have many specialized features, e.g., a phragmoplast-like cytokinesis, presence of plasmodesmata and multilayered structures (Leliaert et al., 2012), and also commonly form lichens (Nelsen et al., 2011). Other members of the Ulvophyceae, e.g., *Desmochloris* have been described from African soils (Darienko et al., 2009).

Streptophyta are comprised of the Charophytes, a paraphyletic assemblage of freshwater algae, and the land plants (Leliaert et al., 2012). The Charophytes contain only around 100 genera of algae, and are therefore a far less diverse group than the Chlorophytes and Prasinophytes, which contain at least several hundred genera (McCourt et al., 2004; Leliaert et al., 2012). The Streptophyta include six morphologically distinct classes: the flagellate Mesostigmatophyceae; the sarcinoid Chlorokybophyceae; the filamentous (unbranched) Klebsormidiophyceae; the Zygnematophyceae, characterized by their ability to reproduce sexually by conjugation; and two morphogenetically complex groups, the multicellular Charophyceae and the Coleochaetophyceae. Terrestrial forms are known in the Chlorokybophyceae (Lewis and McCourt, 2004), the Klebsormidiophyceae (e.g., Karsten et al., 2010; Karsten and Holzinger, 2012), the Zygnematophyceae (e.g., Lewis and McCourt, 2004; Holzinger et al., 2009, 2010) and the Coleochaetophyceae (Graham et al., 2012). A detailed phylogenetic analysis of the Zygnematophyceae was given by McCourt et al. (2000) and Hall et al. (2008), and the phylogenetic relationships of the Klebsormidiophyceae have been investigated by Rindi et al. (2008, 2011); Rindi (2011) and Mikhailyuk et al. (2008). Škaloud and Rindi (2013) suggested an ecological differentiation of cryptic species of *Klebsormidium*.

All streptophyte algae are adapted to fresh water, which allowed them to colonize moderately moist habitats in the proximity of water and from there move gradually to dry land (Becker and Marin, 2009). These authors suggested that the reduction of vegetative desiccation tolerance in advanced Streptophyta made it necessary to develop mechanisms for living on land, e.g., to retain water in the plant (such as a cuticular leaf surface). Moreover, it is possible that the loss of vegetative desiccation tolerance led to the development of water-regulating structures such as xylem elements to transport water, or stomata to regulate transpiration, which are typical for homoiohydric plants.

## EFFECTS OF DESICCATION ON PHOTOSYNTHESIS AND RESPIRATION

**FIGURE 3:**
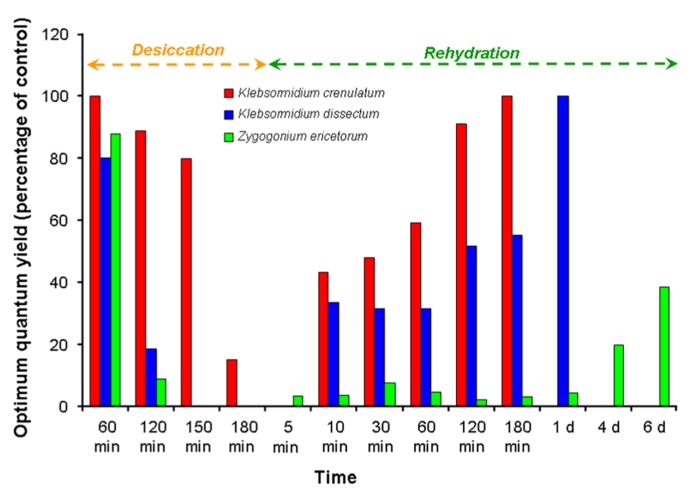
**The effect of desiccation and rehydration on the optimum quantum yield (Fv/Fm) in *Klebsormidium crenulatum* and *Klebsormidium dissectum* isolated from an alpine biological soil crust, and in *Zygogonium ericetorum* collected from an adjacent intermittent streamlet (data adapted from Karsten et al., 2010; Karsten and Holzinger, 2012; Aigner et al., 2013)**.

Dehydration suppresses photosynthesis in green algae (Häubner et al., 2006; Gray et al., 2007; Karsten et al., 2010; Karsten and Holzinger, 2012). A representative example is given in **Figure 3**, which shows the photosynthetic activity under controlled desiccation and rehydration conditions in two aeroterrestrial *Klebsormidium* species from an alpine biological soil crust, together with a semiterrestrial *Zygogonium ericetorum* collected from a nearby intermittent streamlet (data are adapted from Karsten et al., 2010; Karsten and Holzinger, 2012; Aigner et al., 2013). The activity profiles were species-specific, and although both *Klebsormidium* species fully recovered after rehydration, *Zygogonium* showed extensive damage. In *Trentepohlia odorata* (Ulvophyceae) the photosynthesis rates were reduced by low levels of air humidity, and hence changed during the day, with a maximum in midmorning and then decreasing (Ong et al., 1992; **Figure 2D**). Green algal biofilms on tree bark showed a pronounced desiccation tolerance (up to 80 days) regarding their photosynthetic activity, i.e., the kinetics and degree of photosynthetic recovery were faster after shorter periods of dehydration (Lüttge and Büdel, 2010). Under controlled laboratory conditions, unicellular green algae from biological soil crusts in the desert can survive for at least 4 weeks (Gray et al., 2007). In this study, desiccation-tolerant desert algae and closely related aquatic relatives were exposed to dry conditions. The survival and activity rates were investigated in the Chlorophycean genera *Bracteacoccus* sp., *Scenedesmus rotundus*, and *Chlorosarcinopsis* sp., and the Trebouxiophycean genera *Chlorella* sp. and *Myrmecia* sp. Recovery of photosynthetic quantum yield (Fv/Fm) was much better in the desert lineages than in their aquatic relatives. In addition, the desert algae survived desiccation for at least 4 weeks when desiccated in darkness, and recovered to high levels of photosynthetic quantum yield (Fv/Fm) within 1 h of rehydration in darkness. When desiccation was accompanied by irradiation, the recovery rate was drastically reduced, a still-unexplained phenomenon (Gray et al., 2007).

Because in the dehydrated state photosynthesis is usually completely blocked, any further excitation energy absorbed cannot be used for electron transport, and hence may result in photoinhibition or even photodamage (Wieners et al., 2012). For cyanobacteria, lichens, green algae, and mosses, various desiccation-sensitive sites in the photosynthetic apparatus have been reported: the photosystems, particularly photosystem II (PSII) with its oxygen-evolving complex, the ATP-generation, and carbon assimilation processes (Allakhverdiev et al., 2008). Photoinhibition is mainly caused by damage to the D1 protein of PSII. In green algae, photoinhibition, in principle, occurs continuously while the cells are exposed to light, and the damage is continuously repaired, which consists of degradation and *de novo* synthesis of the D1 protein, followed by activation of the reaction center. Because of this rapid repair, most PSII reaction centers are always fully functioning, even under high light conditions. However, desiccation may limit the supply of carbon dioxide for use in carbon fixation, which decreases the rate of repair of D1 protein in PSII by direct inactivation of the translation machinery (Takahashi and Murata, 2008). Consequently, not photodamage, but rather the loss of the repair capacity is the main mechanism for depression of photosynthesis in green algae during desiccation. Interrupting carbon fixation results in the creation of reactive oxygen species (ROS), which in turn not only block the biosynthesis of PSII proteins, but also biochemically affect nucleic acids and polyunsaturated fatty acids (lipid peroxidation).

A proteomic study on a desiccation-tolerant grass found that as photosynthetic activity is blocked during dehydration, photosynthesis proteins also generally decrease to prevent ROS formation (Oliver et al., 2011). Recent proteomic analyses of the lichen green alga *Asterochloris erici *showed that dehydration caused an increase in the relative abundance of only a few proteins (Gasulla et al., 2013a). The authors reported that although they could not identify the proteins involved in the light reactions of photosynthesis, dehydration led to a decrease in proteins associated with the Calvin cycle, indicating a reduction in carbon fixation. However, *A. erici* is still able to maintain photosynthesis at below 10% relative water content (Gasulla et al., 2009), so presumably the loss of certain proteins does not preclude the possibility that carbon fixation could occur during drying (Gasulla et al., 2013a). These data support the conclusion that the desiccation tolerance of *A. erici* is achieved by constitutive mechanisms. Alternative pathways to eliminate harmful energy from the photosynthetic apparatus have been described in *Ulva* sp., and involve a PS I-driven cyclic electron flow, which has been found to contribute to desiccation tolerance (Gao et al., 2012).

The effect on CO_2_ exchange of desiccation induced by reducing the air humidity has been investigated in the aeroterrestrial green alga *Apatococcus lobatus* (Bertsch, 1966). This species is one of the most abundant taxa in temperate Europe, forming conspicuous biofilms on tree bark and building surfaces (Gustavs et al., 2011). *A. lobatus* consists of globular cells, which often divide in 2 or 3 planes to form irregular, cuboidal cell packets or at later stages, biofilms: these cell packets achieve hydration equilibrium with the vapor pressure of the air only (Bertsch, 1966). Although the most favorable carbon assimilation in this green alga was measured at 97–98% RH, at 90% RH 50% of the maximum CO_2_-uptake still occurred. The lower limit of carbon assimilation was observed at 68% RH (Bertsch, 1966). These data clearly indicate that moisture favors CO_2_ uptake in *A. lobatus*, while liquid water has an unfavorable influence. In *Klebsormidium flaccidum* (De Winder et al., 1990) and lichens (Green et al., 2008), the water content of the organism determines the carbon dioxide supply and hence the photosynthesis rate. Dehydration inhibits photosynthesis if the water content falls below species-specific thresholds; but a high water content (i.e., supersaturation, for example, after rain) typically limits carbon-dioxide diffusion into the cells, also reducing photosynthetic activity. The CO_2_-exchange mechanisms in *Apatococcus* and other aeroterrestrial green algae seem to be well adapted to their terrestrial way of life.

An important methodological approach for studying desiccation effects on green-algal photosynthesis is related to the application of pulse-amplitude-modulation (PAM) fluorometry in conjunction with the saturation pulse method. Chlorophyll a fluorescence originates close to the sites where light energy is transformed into chemically fixed energy. The same excitation states that give rise to fluorescence emission also participate in photochemical energy conversion. These features make Chl fluorescence a unique indicator of photosynthetic performance (Schreiber and Bilger, 1993). PAM studies have proved to be a powerful, non-invasive and rapid tool for the elucidation of fundamental processes in photosynthesis, as well as for ecophysiological investigations such as the application of controlled stress scenarios. In the development of many applications and instrumentation, the model green alga *Chlorella vulgaris* was used.

The PAM approach made it possible to document that lichens and their isolated green-algal photobionts exhibited extreme resistance to high light during dehydration (Lange et al., 2006; Wieners et al., 2012), which indicates that they are able to dissipate energy efficiently as heat. Such non-radiative dissipation has been reported for cyanobacteria, various algae, and mosses, but not for angiosperms (Wieners et al., 2012). Although the underlying molecular mechanisms of this desiccation-induced quenching of chlorophyll a fluorescence are still not understood, they seem to be an essential additional component of photoprotection in aeroterrestrial green algae. Other well-characterized photoprotective mechanisms of photosynthesis include the non-photochemical quenching of excitation energy, which involves the quenching of singlet excited-state chlorophylls via enhanced internal conversion to the ground state, thus harmlessly dissipating excess excitation energy as heat (e.g., Lunch et al., 2013). Non-photochemical quenching is based on conformational changes within the light-harvesting proteins of PSII, resulting in changing pigment interactions, which in turn, cause the formation of energy traps. These conformational changes are mediated by a combination of a transmembrane proton gradient, a light-harvesting protein of PSII, and the activity of the xanthophyll cycle. Much of the progress in the fundamental understanding of these photo physiological processes has come from studies of mutants of the model green alga *Chlamydomonas reinhardtii* (e.g., Niyogi, 1999).

The effect of changing water availability and air humidity on an aeroterrestrial green algal biofilm colonizing a building surface was investigated over several months, using *in situ* fluorescence measurements (Häubner et al., 2006). The photosynthetic activity profile of this biofilm was evaluated using a PAM, correlated with the presence of water condensing on the façade. As solar radiation increased during the day, the water film evaporated, resulting in dehydration and hence inhibition of photosynthesis in the green microalgae. However, recovery of photosynthesis occurred within minutes after artificial moistening (Häubner et al., 2006). Similar high tolerance of dehydration has been described for soil-crust green algae of the genus *Klebsormidium* (De Winder et al., 1990; Karsten et al., 2010; Karsten and Holzinger, 2012). However, the desiccation tolerance of *Klebsormidium* species differs, which can be explained by morphological and structural features (Karsten et al., 2010; Holzinger et al., 2011; Karsten and Holzinger, 2012; see also **Figure 3**). While, for example, *Klebsormidium crenulatum*, isolated from an alpine biological soil crust, forms rather long, strong filaments, sometimes growing in rope-like aggregates that protect against water loss, the co-occurring *K. dissectum* has smaller filaments that easily disintegrate.

While photosynthetic activity during dehydration can easily be followed using chlorophyll fluorescence techniques such as PAM, this approach is not applicable to measurements of respiration. Most respiration studies on green algae were done in the fluid phase using Clark-type electrodes or optodes, but these techniques are not suitable for use in desiccation stress, and hence only a few investigations have examined respiration during dehydration in green algae. The best method to follow respiratory activity during dehydration is the infrared gas analyzer (IRGA), which measures the release of carbon dioxide in the dark, under controlled atmospheric conditions. IRGA investigations have been mainly applied to lichens and their green-algal photobionts (e.g., Sundberg et al., 1999). While photosynthetic carbon-dioxide fixation is the basis for algal survival and development, producing the carbohydrates needed for metabolism, protective strategies and structural components, the subsequent respiratory release of carbon dioxide is related to the energy requirements for cell maintenance, nutrient acquisition and growth (Palmqvist and Sundberg, 2002).

Ecophysiological studies on bryophytes, lichens, and intertidal green macroalgae indicate that photosynthesis and respiration show different responses when these plants become dehydrated (Dilks and Proctor, 1979; Karsten et al., 1991; Sundberg et al., 1999), and that respiration is less sensitive to many stress conditions. One explanation for the different susceptibility of these processes may be related to the structural properties of chloroplasts and mitochondria. While chloroplasts easily swell or shrink depending on the external water potential, with consequences for the thylakoid fine structure and hence its function as the locality of the photosynthetic electron transport chain, the ultrastructure of the mitochondrial cristae is much less affected (Kirst, 1990).

As a consequence of reduced or completely inhibited photosynthetic and respiratory activity, other physiological processes such as growth or motility of green algae are also affected. From an ecological perspective, growth is the most relevant physiological process to describe the performance of green algae in their habitat, because it integrates all positive and negative environmental effects on the organism and hence reflects the acclimation potential (Gustavs et al., 2009). If marine green macroalgae are exposed to drying during low tides, growth is normally completely inhibited. The physiological strategy is to cope with and survive this stress condition through maximum reduction of all metabolic activities.

## EFFECTS OF DESICCATION ON BIOCHEMISTRY AND MOLECULAR BIOLOGY

Physiological constraints caused by dehydration in green algae have been investigated mainly in relation to photosynthesis (see above), and hence far less is known about the molecular and cellular changes that accompany water loss.

Desiccation leads to oxidative stress (Scheibe and Beck, 2011). The generation of ROS and their destructive effects have been reviewed extensively, e.g., in lichen desiccation tolerance (Kranner et al., 2008). Oxygen, a byproduct of photosynthesis, is the basis of all aerobic life, playing an essential role in respiration. However, oxygen is also able to form free radicals and to participate in many oxidative chemical reactions (Abele, 2002). Free radicals are atoms or molecules with an unpaired electron. This unpaired electron is readily donated, and as a result, most free radicals are highly reactive. Oxygen radicals include singlet oxygen (^1^O_2_), superoxide $O_{2}^{•-}$, and the hydroxyl radical (^·^OH), and together with hydrogen peroxide (H_2_O_2_), they are chemically aggressive and hence are termed ROS. Dehydration enhances the formation of ROS, and therefore biochemical systems that effectively prevent their formation or scavenge them when formed are essential for the survival of green algae (Kranner and Birtic, 2005). ROS are the most likely source of damage to nucleic acids, proteins, and lipids. Particularly, the hydroxyl radical is extremely reactive, and it easily hydroxylates, for example, the purine and pyrimidine bases in DNA, thus enhancing mutation rates (Halliwell, 1987). ROS damage to proteins is mediated by configuration changes, mostly by oxidizing the free thiol residues of cysteine.

Conspicuous shifts in amino-acid composition have been observed during Streptophyte transition to land (Jobson and Qui, 2011). These authors found that within the Streptophyta there is an increased utilization of charged amino acids, which was considered an important biochemical strategy to maintain protein hydration during desiccation because the proteome struggles to retain adequate cytoplasmic solute concentrations. Protection from desiccation is provided by charged amino acids (Asp, Glu, Arg, and Lys), particularly with hydrophilic EKR residues, which are polar side chains deemed important for protein thermostabilization (Haney et al., 1999). The frequency of these EKR residues in the plastid proteome was significantly higher in bryophytes than in algae (Jobson and Qui, 2011). Among streptophytic green algae, the aeroterrestrial genus *Chlorokybus* exhibited higher concentrations of positively charged amino acids (HKR; Glu, Arg, Lys) compared to their closely related freshwater genus *Mesostigma* (Jobson and Qui, 2011).

Besides the changes in amino acid composition, to our best knowledge, only one study conducted a proteomic analysis upon desiccation in the Trebouxiophycean green alga *Asterochloris* (Gasulla et al., 2013a). The effects of either slow (5–6 h) or rapid (<1 h) desiccation and rehydration after 24 h were investigated by 2D difference gel electrophoresis followed by liquid chromatography-tandem mass spectroscopy (LC-MS/MS). Desiccation increased the abundance of only 11–13 proteins, which was reported to be independent of the drying rate (Gasulla et al., 2013a). The altered proteins are involved in glycolysis, cellular protection, cytoskeleton, cell cycle and degradation. Transcripts of five *Hsp90* and of *β-tubulin* genes accumulated at the end of the dehydration process (Gasulla et al., 2013a). Interestingly, although ultrastructural changes were observed (see below), no major changes occurred in the proteome (Gasulla et al., 2013a).

The role of low-molecular-weight carbohydrates is discussed below in the section “Biochemical protection.” The lipid composition also changes during desiccation, as recently demonstrated in resurrection plants (e.g., Gasulla et al., 2013b). In these plants, the lipid composition underwent major changes, including the removal of monogalactosyldiacylglycerol from the thylakoids (Gasulla et al., 2013b). While compositional changes of lipids in microalgae (*Chlorella vulgaris*) under nitrogen depletion have been addressed on a transcriptomic level (Guarnieri et al., 2011), the consequences of desiccation to the lipid composition in green algae are still unstudied.

## EFFECTS OF DESICCATION ON STRUCTURE AND ULTRASTRUCTURE

**FIGURE 4:**
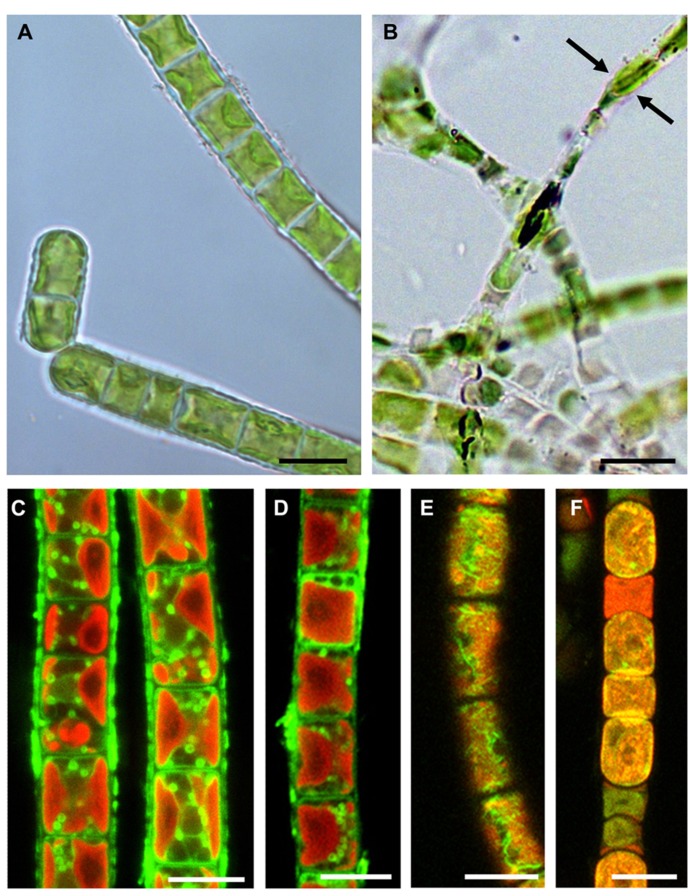
**Comparisons of hydrated and experimentally desiccated cells of the streptophytic green alga *Klebsormidium crenulatum* observed by light- (A,B) and confocal laser scanning microscopy (C–F).** **(A)** cells form liquid culture, **(B)** cells desiccated for 1 day at ~5% RH and observed in immersion oil, note the loss of diameter (arrows), **(C)** mitochondrial staining in hydrated cells, **(D)** mitochondrial staining in 1-day desiccated (~5% RH) cells, **(E)** F-actin staining in hydrated cells, **(F)** severely damaged F-actin in 1-day desiccated (~5% RH) cells. Bars 10 μm (reprinted from Holzinger et al., 2011 with permission from the Phycological Society of America).

Dehydration of green algae leads primarily to a shrinkage process. *Klebsormidium *cells are capable of reducing their diameter to ~50–60% of the original value (**Figures 4A,B**; Holzinger et al., 2011; Karsten and Holzinger, 2012). Cytological alterations may occur to different extents and can be seen after staining the mitochondria (**Figures 4C,D**) or the F-actin cytoskeleton, through the confocal laser scanning microscope (**Figures 4E,F**; Holzinger et al., 2011).

**FIGURE 5:**
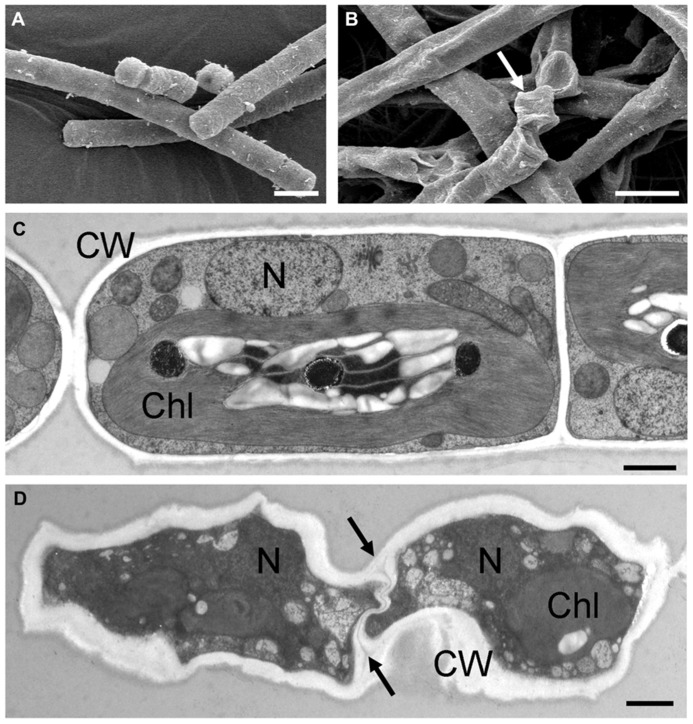
**Comparisons of the ultrastructure of hydrated and experimentally desiccated cells of the streptophytic green alga *Klebsormidium*.** **(A)** hydrated cells from liquid culture, observed by scanning electron microscopy, **(B)** 7-day desiccated (~5% RH) cells, with severely damaged cell walls (arrow), **(C)** transmission electron micrograph of high-pressure frozen freeze-substituted cells from liquid culture, **(D)** desiccated cells (1 day, ~5% RH), cytoplasm appears electron-dense with nucleus and chloroplast still visible, cell walls are irregularly shaped and the cross-walls show an undulating structure (arrows). Bars **(A,B)** 10 μm, **(C,D)** 1 μm. Chl, chloroplast; CW, cell wall; N, nucleus (**A,B** reprinted from Holzinger et al., 2011 with permission of the Phycological Society of America; **C,D** reprinted from Karsten and Holzinger, 2012 with permission from Springer Science and Business Media).

Only a few publications have dealt with ultrastructure as a response to desiccation in green algae (e.g., Morison and Sheath, 1985; Hoppert et al., 2004; Holzinger et al., 2010, 2011; Karsten and Holzinger, 2012; Gasulla et al., 2013a) or osmotic water loss (e.g., Affenzeller et al., 2009; Darehshouri and Lütz-Meindl, 2010; Kaplan et al., 2012, 2013). This is largely due to methodological difficulties in attempting to observe the effects of desiccation at the ultrastructural level. Desiccated stages can easily be investigated in a scanning electron microscope, but only the damage to the cell walls is visible, in comparison to hydrated control cells (**Figures 5A,B**). In contrast, chemical fixation of naturally desiccated samples for transmission electron microscopy (TEM) usually results in rather poor preservation of the ultrastructure (e.g., Morison and Sheath, 1985; Hoppert et al., 2004; Holzinger et al., 2010). Most of the samples investigated were collected from the field and represented permanent stages adapted to withstand unfavorable conditions (e.g., Morison and Sheath, 1985; Hoppert et al., 2004). After chemical fixation, mostly thick cell walls were visible, but many details of the ultrastructure could not be recognized. In contrast, upon experimental desiccation of cultured *Klebsormidium* cells, high-pressure freeze-fixation gave better results and allowed the depiction of ultrastructural details in desiccated cells (**Figures 5C,D**; Holzinger et al., 2011; Karsten and Holzinger, 2012). Overall the cytoplasm appeared extremely dense, with the major organelles, the nucleus and the chloroplast clearly visible. Within the chloroplasts, the number of plastoglobules was increased after desiccation, demonstrating the capability of reorganization during the desiccation process. A similar increase of plastoglobules was observed in the desiccation-tolerant moss *Syntrichia* (Fernández-Marín et al., 2013). However, increased numbers of plastoglobules were also observed after various stresses, including light stress (e.g., Holzinger et al., 2004; Holzinger and Lütz, 2006), so that this cannot be regarded as a desiccation-specific response.

Plastoglobules are lipoprotein subcompartments of the chloroplast, which were shown to be permanently coupled to thylakoid membranes (Austin et al., 2006). They contain biosynthetic and metabolic enzymes (Austin et al., 2006; Ytterberg et al., 2006), which may also be important for desiccation tolerance. The regulated “melting” of the thylakoid membranes to plastoglobules might somehow contribute to light protection during desiccation (Fernández-Marín et al., 2013).

Substantial destruction of the actin filament system was another consequence of desiccation (**Figures 4E,F**), as investigated by phalloidin staining and confocal laser scanning microscopy (Holzinger et al., 2011). The changes in the ultrastructural appearance of the cytoplasm were very similar to those seen after experimental dehydration by hypertonic sorbitol solution (Kaplan et al., 2012). The key differences after desiccation were the strongly undulating cross-cell walls, resulting from shrinkage (Holzinger et al., 2011; Karsten and Holzinger, 2012).

Biochemical investigations of the cell wall of *Klebsormidium* became available only recently, and demonstrated that the cell wall lacks most of the epitopes and glycosidic linkages that are typical for embryophyte walls (Sørensen, 2011). The cell wall is relatively poor in cellulose but contains callose instead, which explains its outstanding flexibility upon desiccation (Holzinger et al., 2011; Karsten and Holzinger, 2012). Flexible cell walls might be a key structure contributing to the desiccation tolerance of *Klebsormidium*. While the ultrastructure of the cytoplasm appeared similar after osmotic dehydration, the cell walls remained intact and a large periplastic space became visible in *Klebsormidium *(Kaplan et al., 2012). Similar observations have been made in *Closterium *(Domozych et al., 2003) and in *Zygnema *(Kaplan et al., 2013).

Another approach was followed by Gasulla et al. (2013a), who found ultrastructural changes in cells of *Asterochloris erici* after 3 h of rehydration following fast (<60 min) or slow (5–6 h) desiccation. While slow dehydration resulted in an increasing number of lipid bodies together with a reduction in size, the quantity of starch deposits located within the chloroplasts and electron-dense deposits in the chloroplasts increased (Gasulla et al., 2013a). In the slowly dried and rehydrated cells, the plasma membrane still remained slightly retracted from the cell wall. In contrast, rapidly dried cells of *Asterochloris* following rehydration clearly exhibited a degenerate ultrastructure. The cytoplasm was highly vacuolated and filled with lipid bodies, the cytoplasm and the chloroplasts still appeared shrunken, thylakoids were swollen or fused, and numerous starch deposits were visible (Gasulla et al., 2013a). Rapidly dried *Asterochloris* cells exhibited extensive plasmolysis and cytolysis. However, even with this damage, the cells survived the dehydration treatment. The possible flexibility of the cell walls was not investigated in the rehydrated cells.

## EFFECTS OF DESICCATION ON THE ECOLOGY OF GREEN ALGAE

Although the primary production of aeroterrestrial green algae has not been studied, it is reasonable to assume that dehydration will have a strong negative effect, because dehydration leads to a decreased in photosynthetic activity. Because most lichens and their photobionts grow relatively slowly, their primary productivity is fairly low in most ecosystems. Nevertheless, a yearlong field study of a lichen in the Negev Desert showed that this cryptogamic organism was metabolically active on most days of the year. Dewfall and sporadic rainfall caused such favorable conditions that on 68% of all days a positive, but moderate carbon balance could be determined (Kappen et al., 1979). Along with lichens, bryophytes and microfungi, mainly green algae and cyanobacteria form a joint matrix by gluing together soil particles and themselves, thereby forming a productive microbial biomass in many arid regions (Belnap and Lange, 2001). The resulting biological soil crusts have important, multi-functional ecological roles in primary production, nitrogen cycling, mineralization, water retention, stabilization of soils, and dust trapping (Evans and Johansen, 1999; Reynolds et al., 2001; Gray et al., 2007; Castillo-Monroy et al., 2010; Elbert et al., 2012). These essential ecological functions for arid and semi-arid regions are under threat, because a recent study of global terrestrial primary production indicated that the past decade (2000–2009) has been the warmest since instrumental measurements began, which led to reduction in regional terrestrial primary production due to large-scale droughts and a drying trend in the Southern Hemisphere (Zhao and Running, 2010). These climatic changes will of course also affect aeroterrestrial green algae, whether free-living or in association with biological soil crusts or as photobionts with lichens.

Most tidal-influenced rocky shores show a conspicuous zonation of seaweeds, which are segregated into horizontal bands across the vertical rock surface (Benson, 2002). The highest is the so-called supralittoral zone, which is mainly exposed to the atmosphere and only influenced by spray or splashing waves during storm events at high tides. In this zone, typical green macroalgae of the genera *Prasiola* or *Rosenvingiella* occur under almost fully terrestrial conditions, and thus are exposed to drying for long intervals (Rindi et al., 1999; Holzinger et al., 2006). *Prasiola* species in polar regions often grow in association with penguin or seagull colonies, even several meters above sea level, because they are extremely nitrophilous and prefer habitats rich in mixed excreta and feces of birds (Rindi et al., 1999).

Just below the supralittoral zone is the so-called upper intertidal zone, which is covered with seawater only during high tides. Here, many filamentous or foliose green macroalgae such as members of the opportunistic genera *Ulothrix*, *Ulva*, *Urospora*, etc. often form green belts. These algae are also influenced by drying at low tides, but more on a diel scale. The intertidal zone represents an interface between the terrestrial and marine environment, which may vary with season of the year and geography. Typical for such ecotones are edge effects, where certain species spend most or all of their time in this transitional habitat. Dehydration is the main environmental factor in the supralittoral and high intertidal zones, and the green macroalgae living in these zones are exposed regularly to air, yet still survive. These plants lose considerable amounts of water when exposed to the atmosphere and sun, but for example, *Urospora* species can survive more than 20 days of air exposure. However, the intertidal zone and the zonation of seaweeds are controlled not by drying alone, but by a combination of abiotic and biotic factors. In addition, microhabitats such as crevasses, underneath boulders, or beneath the canopy of overlying macroalgae can offer protection against dehydration.

## PROTECTIVE STRATEGIES

### AVOIDANCE OF DESICCATION

One strategy of aeroterrestrial and aquatic green algae against desiccation is to avoid dehydration by self-protection. Under natural conditions, aeroterrestrial filamentous green algae such as *Klebsormidium *can form multi-layered mat-like structures on top of or interwoven with the upper millimeters of soil, which contribute to a high degree of self-shading and reduced loss of water from individual filaments within such a population. Mat formation has also been observed in Arctic *Zygnema* sp. (Holzinger et al., 2009; Pichrtová et al., 2013), and contributes to desiccation tolerance in field-collected *Zygogonium ericetorum* in the Alps (Holzinger et al., 2010; Aigner et al., 2013). Gray et al. (2007) suggested that in biological soil crusts of North American deserts, the green algae may occupy microenvironments within the crust matrix, where they are protected from damaging light levels and exposure to drying atmosphere. These data clearly indicate that single green-algal cells, which are closely associated with other algal cells in an aggregate (e.g., young *Apatococcus*), colony (e.g., *Coccomyxa*), biofilm (e.g., mature *Apatococcus*) or biological soil crust (e.g., *Klebsormidium*) are much better protected against dehydration than are algal cells living solitarily. Additional factors that contribute to avoidance or at least retardation of water loss include a low algal surface-to-volume ratio and morphological features such as thick cell walls and mucilage layers (e.g., *Prasiola*; Jacob et al., 1992). Extracellular polysaccharides (EPS) are critical in desiccation tolerance of cyanobacteria and have beneficial effects on desiccation tolerance in the green alga *Chlorella *sp. (Knowles and Castenholz, 2008). Under exposed conditions, macroalgal canopies of *Ulva* sp. in the upper intertidal zone in southern Spain form sheet-like, multiple-layered structures in which the top layer usually bleaches due to strong solar radiation, desiccation, and other abiotic stresses, thereby providing photoprotection and moisture for subcanopy thalli (Bischof et al., 2002).

### AVOIDANCE THROUGH SPORES AND PERMANENT STAGES

Factors leading to spore formation and germination have been reviewed extensively (Coleman, 1983; Agrawal and Singh, 2000; Agrawal, 2009, 2012). Desiccation events have a major impact on the transition from the vegetative to different forms of permanent stages such as akinetes, zygospores, oospores, or cysts (e.g., Starr, 1955; Evans, 1958).

The formation of akinetes has been observed frequently in field-collected *Zygnema* sp., and is likely involved in its desiccation tolerance. These akinetes are older cells that have accumulated large amounts of lipids and phenolic substances in the cytoplasm, while the vacuoles are drastically reduced (Holzinger et al., 2009; Pichrtová et al., 2013). Similar observations were made in the closely related *Zygogonium ericetorum* collected in high-alpine habitats (Holzinger et al., 2010; Aigner et al., 2013). Recently, the phenolic compounds were further characterized, and these also likely contribute to UV tolerance of these cells (Aigner et al., 2013). Genkel and Pronina (1979) reported that *Zygnema* *stellinum *became greenish-brown, entering a resting state (akinetes), which allowed them to desiccate during summer, before they formed parthenospores for overwintering. Some cell walls of zygospores of *Zygnema*, described by Stancheva et al. (2012), have a purple cell wall that possibly contains similar substances to those described by Aigner et al. (2013) for *Zygogonium*. Phenolic compounds in brown algae include phlorotannins, which contribute considerably to protecting sensitive life stages from irradiation (e.g., Holzinger et al., 2011).

Hypnoblasts, as described in the snow alga *Chlamydomonas nivalis* by Remias and Lütz (2005), are particularly well prepared to tolerate unfavorable conditions such as freezing-induced water stress. However, when considering the time needed for germination of spores, tolerating desiccation in the vegetative state is substantially faster, and organisms capable of this strategy have a clear advantage. For example, in glaciers, where liquid water may be only intermittently available due to freezing, only vegetative states of the Zygnematophyceae *Mesotaenium *and *Ancylonema *have been found (Remias et al., 2009, 2012).

### PHYSIOLOGICAL PROTECTION

As discussed above, the physiological mechanism that is most sensitive to dehydration is photosynthesis, and hence efficient control of light absorption and energy distribution in the photosynthetic apparatus during dehydration seems to be most important to reduce or prevent photoinhibition. Photoinhibition can be distinguished as dynamic and chronic forms, the latter representing irreversible photodamage. In contrast, dynamic photoinhibition is a reversible, controlled down-regulation of photosynthesis in the light under different stress conditions (Hanelt, 1998). The main mechanism of dynamic photoinhibition is non-photochemical quenching of excitation energy absorbed by PSII, through harmless dissipation as heat. This photoprotective strategy compensates for at least “short-term” stress conditions, such as high light and drying at low tide at noon. Dynamic photoinhibition has also been confirmed for several species of desert and aquatic green algae (Lunch et al., 2013). These authors found that although photoprotective mechanisms in green algae are similar in principle, they exhibit lineage-specific modifications. De-epoxidation of xanthophyll-cycle pigments paralleled light-induced changes in non-photochemical quenching for species of Klebsormidiophyceae and Trebouxiophyceae, but not Zygnematophyceae, indicating that the pigments involved can contribute to photoprotection, although to different degrees in different lineages (Lunch et al., 2013). Recently, Kosugi et al. (2013) found that arabitol provided by the fungal partner of the lichenized green alga *Trebouxia* sp. enhances the alga’s ability to dissipate excess light energy.

The evolution of photoprotective mechanisms upon land colonization was recently studied by Gerotto and Morosinotto (2013) in more recently evolved streptophycean green algae. The need for photochemical quenching appears to be regulated by different proteins, on the one hand by a light-harvesting complex-like stress-related protein (LHCSR), whereas a photosystem II subunit S protein (PSBS) was detected in Zygnematales, Charales, and Coleochaetales. Moreover, there is a major difference between the glycolate pathway between the Chlorophyta and Streptophyta, as investigated in *Mougeotia scalaris* (Charophyta) and *Eremosphaera viridis* (Chlorophyta) by Stabenau and Winkler (2005). *Eremosphaera viridis* does not possess peroxisomes as found in angiosperm leaves, and all reactions (glycolate oxidation and ATP generation) are performed exclusively in the mitochondria.

In addition to the protection of molecular components of the photosynthetic apparatus against dehydration, the thylakoids may also be partially or completely protected by the presence of low-molecular-weight carbohydrates during water stress (Santarius, 1973). Responses similar to those that occur during water stress may be seen during cold acclimation in *Klebsormidium* (Elster et al., 2008). Large amounts of sucrose have been detected in *Klebsormidium flaccidum* because of cold acclimation (Nagao et al., 2008), and the occurrence of sucrose-phosphate phosphatase with altered domains compared to land plants has been reported in the same species (Nagao and Uemura, 2012). Membrane stabilization depends on the concentration of sugars and their molecular mass, so that the trisaccharide raffinose is more effective than the disaccharide sucrose and the monosaccharide glucose (Santarius, 1973). Since green algae can synthesize and accumulate an array of chemically different low-molecular-weight carbohydrates, especially under conditions of low water potential (Karsten et al., 2007), this strategy can be regarded as important for protecting the photosynthetic membranes.

### BIOCHEMICAL PROTECTION

In addition to the effects of desiccation on photosynthesis and the protection or *de novo* biosynthesis of the photosynthetic apparatus, various additional protective biochemical mechanisms have been suggested. Non-reducing low-molecular-weight carbohydrates such as sucrose and trehalose protect not only thylakoids, but also other membranes and proteins from dehydration damage (Santarius, 1973). Particularly trehalose is strongly involved in desiccation tolerance in many biological systems (Yancey, 2005). During dehydration, this disaccharide may bind to biomolecules and membranes by replacing water and thus maintaining their basic structure. Trehalose forms a glass-like state (=supercooled liquid with extreme viscosity) under dry conditions, and glass formation (vitrification) is considered to be a prerequisite for structural stabilization of dried cytoplasm (Buitink et al., 2002). However, in yeast, trehalose was found to be neither necessary nor sufficient for desiccation tolerance (Ratnakumar and Tunnacliffe, 2006). Moreover, this widely distributed sugar as well as other soluble carbohydrates were detected only in trace amounts in *Klebsormidium* (Kaplan et al., 2012), which suggests that this desiccation-tolerant alga uses different protection mechanisms.

The hydration of proteins by water molecules is important in maintaining their three-dimensional structure and consequently their function. While morphological structures may prevent or delay water loss, the biosynthesis and accumulation of high concentrations of organic osmolytes such as polyols, betaines, proline etc. are also thought to contribute to desiccation tolerance (Oren, 2007), because high concentrations of these compounds generate low water potentials in the cytoplasm without incurring metabolic damage (Yancey, 2005; and references therein). For these organic compounds that are tolerated at high intracellular concentrations, the term “compatible solute” was introduced by Brown and Simpson (1972).

Polyols perform multiple functions in metabolism; in addition to their roles as organic osmolytes and compatible solutes, they can also act as antioxidants, heat protectants (stabilization of proteins), and rapidly available respiratory substrates (energy supply for a maintenance metabolism under stress and for repair processes; Yancey, 2005; Karsten et al., 2007; and references therein). Typical aeroterrestrial green-algal taxa such as *Apatococcus*, *Chloroidium*, *Coccomyxa*, *Prasiola*, *Stichococcus*, and *Trentepohlia* synthesize and accumulate high concentrations of a range of polyols such as glycerol, erythritol, ribitol, arabitol, mannitol, sorbitol, and volemitol (Feige and Kremer, 1980; Gustavs et al., 2010, 2011), and the polyol metabolism forms an integral part of a biochemical protective strategy against water loss. In contrast, marine green macroalgae such as *Acrosiphonia*, *Cladophora*, *Ulothrix*, *Ulva*, and *Urospora* lack polyols, and instead synthesize and accumulate other organic compounds such as sucrose, proline, glycine betaine, or dimethylsulfoniopropionate (DMSP; Kirst, 1990; Karsten, 2012).

Although they differ in their chemical structure, polyols and the other organic solutes in green algae have several features in common: they are highly soluble, have no net charges at physiological pH, and are non-inhibitory at high concentrations (Kirst, 1990; Karsten et al., 1996). The interactions of these compounds with intracellular macromolecules are not completely understood, and several mechanisms have been suggested. Bisson and Kirst (1995) discussed the different models proposed to explain protection of enzyme systems: (I) binding of the solute to the protein, (II) colligative action of the solute, (III) buffering of potentially damaging changes in solution properties, (IV) inhibition of conformational changes resulting in the formation of inter- or intramolecular disulfide bridges, and (V) preferential exclusion of the solute from the protein surface. These models can be grouped into two basic types: (1) those that hypothesize the existence of direct solute-protein interactions, and (2) those that postulate that protein stability is mediated by solute-induced changes in water structure (Roberts, 2005; Yancey, 2005). However, there is little experimental evidence in green algae for any of these models.

Typical antioxidant mechanisms in lichens and their green photobionts include protective enzymes, such as superoxide dismutase, catalase, peroxidases, glutathione reductase, and ascorbate peroxidase, in combination with non-enzymatic substances such as glutathione, α-tocopherol, and ascorbic acid (Kranner et al., 2005; Kranner and Birtic, 2005; Weissman et al., 2005). In addition, some pigments such as the secondary carotenoid astaxanthin, which is formed and accumulated in high concentrations by various green algae such as *Haematococcus* (Borowitzka et al., 1991) or green snow algae (Remias and Lütz, 2005, 2007), exhibit strong antioxidative capabilities. A potent antioxidant system seems to be one of the underlying mechanisms of desiccation tolerance. However, when oxidative processes prevail during “long-term” desiccation, the antioxidant capability may break down, with negative effects on viability when favorable conditions return.

The late embryogenesis abundant (LEA) proteins are biomolecules in plants that protect other proteins from aggregating during dehydration, probably due to conformational changes in transcription factors or integral membrane proteins (Goyal et al., 2005). Additional functions of LEA proteins in protecting DNA and stabilizing cytoskeletal filaments have been suggested (Wise and Tunnacliffe, 2004). The possible occurrence of LEA proteins was demonstrated in the green alga *Chlorella vulgaris*, by the nucleotide sequence of a cDNA clone of the *h*ardening-*i*nduced *Chlorella* (*hiC*) gene (Honjoh et al., 2000). Purified HIC6 and HIC12 proteins were found to be highly effective in protecting a freeze-labile lactate dehydrogenase (LDH) from rabbit muscle. It can be expected that these proteins also protect against desiccation.

### *DE NOVO* BIOSYNTHESIS AND REPAIR

DNA is the only biomolecule in cells that is steadily maintained and repaired, while all other biomolecules such as proteins are degraded in case of damage, followed by *de novo* biosynthesis. DNA repair involves a set of processes by which a cell identifies and corrects damage to the DNA molecules, which is vital to the integrity of the respective genome, and thus to the normal functioning of the genome and the organism. A typical DNA repair enzyme is blue-light controlled photolyase (Beel et al., 2012).

Concerning repair mechanisms, one must be aware that polynucleotides have astonishing stability, as was demonstrated for cyanobacteria (as summarized, e.g., by Lüttge, 2011). DNA and rRNA were remarkably stable for many years in the desiccated state. However, in case of damage, a protein (DdrA) with an affinity for the 3′ ends of “single-stranded” DNA protects these ends from nuclease digestion (Potts et al., 2005). In the cyanobacterium *Gloeobacter violaceus*, a gene with 40% similarity to DdrA was reported. This organism grows in the Limestone Alps and therefore is frequently exposed to desiccation. Similar molecular protective strategies are expected to occur in eukaryotic green algae.

As mentioned above, for optimum function of the photosynthetic apparatus, the D1 protein of PSII plays a key role. The natural turnover of this protein is rather rapid, e.g., with a half-life of only 30 min as described for the cyanobacterium *Synechocystis *(Kanervo et al., 1993). When breakdown dominates biosynthesis, the PSII is inactivated, as often observed in green algae during dehydration. Damaged PSII is repaired through the replacement of damaged D1 by newly synthesized D1 protein. The damaged D1 protein is cleaved by a specific endoprotease, and is degraded by a metalloprotease for removal from PSII. At this point, it seems likely that PSII might be disassembled to some extent and then D1-depleted PSII is repaired by the introduction of newly synthesized D1 protein. After PSII has been reassembled, with the incorporation of the newly synthesized D1 protein, the complex is fully functioning again (Allakhverdiev and Murata, 2004). Upon rehydration, *de novo* biosynthesis of D1 protein occurs rapidly, which allows a rapid recovery of the photosynthetic quantum yield (Fv/Fm) in green algae (e.g., Karsten et al., 2010; Karsten and Holzinger, 2012). Mulo et al. (2012) compared the strategies for psbA gene expression, encoding the D1 protein, in cyanobacteria, green algae and vascular plants. In *Chlamydomonas reinhardtii* the expression of the psbA gene is strongly regulated by mRNA processing, particularly at the level of translation initiation. Replacement of damaged D1 protein requires several auxiliary proteins, and many of these chaperones are conserved in both, prokaryotes and eukaryotes (Mulo et al., 2012).

## CONCLUSION

In the present review, we summarize the current knowledge of desiccation effects in green algae. Since the review of desiccation tolerance of green algae by Holzinger (2009), extensive information on this topic has been acquired through physiological and ultrastructural investigations. It is clear that several distinct phylogenetic lineages of green algae are capable of desiccation tolerance in their natural environments. These algae are able to survive desiccation conditions through different strategies: (I) avoidance by intrinsic mechanisms to retain water, e.g., by maintaining high osmotic values in the vegetative state; (II) tolerance of unfavorable conditions by the formation of permanent stages (which is considered to be a very effective but slow process); (III) true desiccation tolerance by survival of severe water loss in the vegetative state. This last mechanism, which is extremely important for the distribution of green algae, has been investigated experimentally in several lichen-forming algae from the Ulvophyceae, and in the Klebsormidiophyceae and Zygnematophyceae from the streptophytic lineage. It is remarkable that desiccation tolerance has evolved several times, but is completely lost in some morphologically advanced Streptophyta (e.g., Charophyceae). However, recent studies on the Zygnematophyceae have suggested this represents the sister group to land plants (e.g., Wodniok et al., 2011; Timme et al., 2012). While desiccation-tolerance mechanisms were likely advantageous for the transition of algae from the aquatic to the terrestrial lifestyle, these mechanisms have not been established permanently in land plants. This failure to establish can also be viewed in the context of the costs of vegetative desiccation tolerance, as metabolic rates in desiccation-tolerant organisms are low compared to the metabolisms of desiccation-sensitive plants (Oliver et al., 2000). For plants to succeed permanently on land, the development of homoiohydric mechanisms of regulating the water status by water transport (e.g., development of xylem vessels) and protection against evaporation (e.g., formation of cuticles) was the most successful strategy. Presently, only a very small proportion of angiosperms (the resurrection plants) are desiccation-tolerant in their vegetative organs (e.g., Gaff and Oliver, 2013).

Because of the lack of available genome information, it is still difficult to address the molecular mechanisms involved in the desiccation tolerance of green algae. In addition, only a few studies have used modern approaches such as metabolomics or proteomics to examine these organisms. Therefore, the determination of the genomes of more types of aeroterrestrial green algae is urgently needed to reach a fundamental understanding of desiccation stress responses.

## Conflict of Interest Statement

The authors declare that the research was conducted in the absence of any commercial or financial relationships that could be construed as a potential conflict of interest.
